# Harboring potential of enzymatic antioxidants in sweet potato [*Ipomoea batatas* (L.) Lam.] nodal cultures under *in vitro* NaCl-mediated salinity stress conditions

**DOI:** 10.3389/fpls.2026.1740941

**Published:** 2026-02-26

**Authors:** Ananya Mishra, Madhumita Dasgupta, Sansuta Mohanty, Pradyumna Tripathy, Hanume Gowda Krishnappa, Satyapriya Singh, Vijay Bahadur Singh Chauhan, Rameshkumar Arutselvan, Bibhuti Bhusan Sahoo, Manas Ranjan Sahoo

**Affiliations:** 1 Department of Vegetable Science, College of Agriculture, Odisha University of Agriculture and Technology, Bhubaneswar, India; 2 Department of Molecular Biology and Biotechnology, Institute of Agricultural Sciences, Siksha O Anusandhan, Deemed to be University, Bhubaneswar, Odisha, India; 3 ICAR Research Complex for Eastern Region, Farming System Research Centre for Hill and Plateau Region, Ranchi, India; 4 ICAR–Central Tuber Crops Research Institute, Regional Centre, Bhubaneswar, India; 5 Central Horticultural Experiment Station, ICAR–Indian Institute of Horticultural Research, Bhubaneswar, India; 6 Regional Research and Technology Transfer Station (RRTTS), Bhawanipatna, Odisha University of Agriculture and Technology, Bhubaneswar, India; 7 ICAR–Central Tuber Crops Research Institute (ICAR–CTCRI), Thiruvananthapuram, India

**Keywords:** antioxidative enzymes, *in vitro* screening, salinity, stress tolerance, sweet potato

## Abstract

Salinity stress hinders the production and productivity of sweet potato worldwide. Stringent selection under *in vitro* salinity conditions would be a nebulous approach to developing stress-tolerant lines. Eight pre-breeding sweet potato genotypes, SP-12, SP-13, SP-23, SP-27, SP-33, SP-42, SP-44, and SP-45, selected from a broad genetic base of 380 germplasms, were evaluated under NaCl-mediated salinity stress conditions using nodal cultures *in vitro*. Sweet potato nodal cultures were raised in Murashige and Skoog (MS) medium with different levels of salinity (0, 50, and 100 mM). The morphological, physiological, and antioxidative enzyme activities under control conditions and salinity stress were assessed. Shoot and root organogenesis under the NaCl-induced MS medium (100 mM) were consistent in genotype SP-13. The antioxidative enzymes increased significantly [0.1-fold in catalase (CAT) to 2.7-fold in ascorbate peroxidase (APX) and guaiacol peroxidase (GPX)] with increasing salinity stress conditions compared to the control across the genotypes. Genotypes SP-13, SP-12, and SP-42 exhibited a higher stress tolerance index (STI) for antioxidative enzymes (AOEs). Pearson’s correlation coefficient (r) matrix revealed a strong integration among the growth parameters and AOEs. Among the antioxidative enzymes, APX (r = 0.74) and dehydroascorbate reductase (DHAR; r = 0.60) showed strong and positive correlations with glutathione reductase (GR). Polyphenol oxidase (PPO) exhibited a strong negative correlation with shoot parameters, including shoot length (r = −0.74) and shoot number (r = −0.71), indicating its association with shoot retardation. Principal component analysis (PCA) and hierarchical clustering indicated that genotype SP-13, followed by genotypes SP-12 and SP-42, is closely related to higher growth performances and better antioxidative enzyme mechanisms. Overall, SP-13, SP-12, and SP-42 performed well, maintaining plantlet growth and AOE properties. The results encourage the rapid screening of the more extensive pre-breeding populations to streamline breeding strategies and develop salinity-tolerant plants.

## 1 Introduction

Salinity represents a significant environmental constraint that adversely affects crop productivity (Shrivastava and Kumar, 2015). Excessive seawater, predominantly Na^+^ and Cl^−^, is a major salt component that hinders essential nutrient uptake and subsequently affects agricultural production and productivity in coastal regions (Ahmed et al., 2020). Salinity is an important factor limiting the growth of most non-halophytic plants, including sweet potato (Villa–Castorena et al., 2003). Sweet potato (*Ipomoea batatas* L. Lam.) is an herbaceous dicotyledonous species and an immensely bio-efficient crop in the family Convolvulaceae. Although the crop originated in Central and South America, it has been widely domesticated in the coastal region of India (Villaba et al., 2024). Salinity stress hinders the production and productivity of sweet potato in the coastal region (Mukhopadhyay and Karisiddaiah, 2014).

Excessive salt inhibits the physiological and biochemical metabolism of crops, resulting in Na^+^ imbalance, osmotic stress, oxidative stress, cellular toxicity, mineral toxicity, etc., ultimately affecting the growth and development of crops (Bhupenchandra et al., 2024). Monovalent cations Na^+^ and K^+^ enter the plant cell through non-selective cation channels (NSCCs) and high-affinity K^+^ (HKT)-type transporters in plants (Munns and Tester, 2008). Salinity creates Na^+^ imbalance by driving excessive passive Na^+^ entry, blocking K^+^ uptake, and overwhelming active Na^+^ extrusion and cellular compartmentalization, leading to cytosolic toxicity and redox environment. Salinity stress induces Na^+^ influx, membrane depolarization, and mitochondrial and chloroplast electron leakage (Hernández, 2019). The process generates reactive oxygen species (ROS) such as superoxide (O_2_^•−^), hydrogen peroxide (H_2_O_2_), and hydroxyl radicals (•OH). HKT transporters are the membrane proteins sensitive to oxidative damage in the plant cell. A plant deploys its potent ROS scavenger mechanisms, such as antioxidative enzymes, to equilibrate ROS overproduction (Deinlein et al., 2014). Antioxidative enzymes scavenge ROS by catalyzing redox reactions and transforming harmful radicals like superoxides into less toxic substances. Superoxide dismutase (SOD) converts O_2_^•−^ to H_2_O_2,_ which is further depleted into water (H_2_O) and molecular oxygen (O_2_) by catalase (CAT) and peroxidases (POX), effectively neutralizing free radicals to prevent cellular damage (Saleem et al., 2024). A clear understanding of the enzymatic antioxidative system is essential for crop selection and deriving stress-tolerant plants (Ashraf and Munns, 2022).

Screening of germplasms is essential to identify genotypes suitable for different abiotic stresses, wastelands, and problematic areas (Dasgupta et al., 2007). *In vitro* screening would provide an efficient and meaningful tool for the rapid genetic evaluation and characterization of genotypes for abiotic stresses, such as salinity and drought tolerance, under a controlled environment in a limited space over a short time span (Sahoo et al., 2018). The plantlets growing *in vitro* exhibited similar responses to abiotic stresses as the field plants without environmental interference. *In vitro* screening through shoot apex and nodal segment culture would provide a systematic, faster, hassle-free, and effective means to identify stress-tolerant plants (Dasgupta et al., 2008). Extensive research has been performed on the salinity tolerance of rice (Rossatto et al., 2017), chickpeas (Rohit et al., 2020), garden peas (Grozeva et al., 2023), groundnuts (Radhakrishnan et al., 2003), tomatoes (Seth and Kendurkar, 2015), potatoes (Magawry et al., 2015), cayenne pepper (Siregar et al., 2020), brinjal (Hannachi et al., 2021), canola (Saleem et al., 2024), and Bermuda grass (Iqbal et al., 2024); however, reports on salinity stress tolerance studies with respect to the antioxidative enzymes in sweet potato are scanty and limited to SOD, CAT, and guaiacol peroxidase (GPX) (Dasgupta et al., 2008).

Plants adapt to harsh environments by deploying avoidance and tolerance mechanisms. Avoidance mechanisms include morphological adjustment and physiological alterations, whereas tolerance mechanisms involve biochemical and molecular strategies to combat stress consequences (Sahoo et al., 2018). Abiotic stress increases the production of ROS, in terms of O_2_^•−^, H_2_O_2_, and •OH, in mitochondria, peroxisomes, and chloroplasts (Apel and Hirt, 2004), which is scavenged by antioxidative enzymes such as superoxide dismutase, peroxidase, and catalase (Mittler, 2017). Therefore, proactive antioxidant machinery is necessary for salt-tolerant plants to effectively eliminate the negative consequences of ROS under salinity and subsequently maintain plant growth and development (Rout and Shaw, 2001). Enzymatic antioxidative properties could be reliable indicators for the quick screening of genotypes for stress tolerance breeding (Devi et al., 2020).

The selection of suitable sweet potato varieties would be a nebulous approach to combat salinity, as soil and water management are practically not feasible (Sapakhova et al., 2023; Bhupenchandra et al., 2024). Selection breeding, which involves understanding tolerance mechanisms under controlled salinity stress conditions, is a rapid, efficient, and robust technique to select suitable genotypes for harsh environments (Dasgupta et al., 2008). Using shoot apex and nodal segment culture for *in vitro* screening would be the most methodical, rapid, and effective way to screen stress-tolerant plants (Dasgupta et al., 2008; Mukherjee, 2001). The present study aimed to assess eight sweet potato pre-breeding lines under *in vitro* NaCl-mediated stress conditions by understanding the antioxidative properties and growth performances from the nodal cultures. The study would help select the pre-breeding lines to augment stringent breeding strategies for salinity tolerance.

## 2 Materials and methods

### 2.1 Experimental site and plant materials

This study was carried out at the Indian Council of Agricultural Research–Central Tuber Crops Research Institute (ICAR–CTCRI), Regional Centre, Bhubaneswar, India. The center is located at latitude 20.24° N and longitude 85.78° E, 45 m above sea level in the southeastern coastal plain zone. Eight sweet potato pre-breeding lines, viz. SP-12, SP-13, SP-23, SP-27, SP-33, SP-42, SP-44, and SP-45, maintained in the gene bank at ICAR–CTCRI national active germplasm sites (NAGS), Bhubaneswar, India, were used as the source materials for the study. The pre-breeding lines were selected from 380 germplasms after rigorous screening and evaluation in pots and field conditions.

### 2.2 Culture medium and NaCl treatments

The culture medium included the Murashige and Skoog (MS) basal medium (Murashige and Skoog, 1962) along with added concentrations of kinetin (2.0 mg L^−1^) and CaCl_2_ (40 mg L^−1^). The media were prepared by dissolving 1-L pre-mix sachets (Himedia, Mumbai, India) in MiliQ water. Various levels of NaCl (0, 50, and 100 mM) were incorporated into the MS basal medium to induce salinity stress conditions. The pH was adjusted to 5.8. The prepared media were autoclaved at 121 °C temperature and 15-lb pressure for 20–30 min and poured (50 mL each) into phyta jars (Tarsons, Kolkata, India). The culture media were stored at room temperature at 25 °C ± 2 °C for 2–3 days to check for any visible microbial growth before *explant* inoculation.

### 2.3 Explant selection and culture conditions

Nodal segments from the sweet potato vines were collected from eight genotypes (SP-12, SP-13, SP-23, SP-27, SP-33, SP-42, SP-44, and SP-45) and processed for inoculation in the NaCl-mediated culture medium *in vitro*. The collected nodal explants were cut into 5–10-mm segments at the nodal joints and washed adequately in running tap water four to five times. Explants were sterilized using a 0.1% Tween-20 for 20 min and washed in running tap water thrice. Explants were further surface-sterilized with 0.1% mercuric chloride for 5 min and washed thoroughly with double-distilled water thrice. The sterile explants were immersed in 70% ethanol for 30–45 seconds, washed thoroughly with double-distilled water thrice, and air-dried inside the laminar airflow. The surface-sterilized explants were inoculated in the previously prepared culture media in phyta jars under aseptic conditions and stored at 22 °C ± 2 °C with a 16/8 h light/dark cycle and a 45 µmol m^−2^ s^−1^ irradiance level provided by cool/white fluorescence tubes with 55%–60% relative humidity (RH) for 6 weeks. Shoot and root proliferation of *in vitro* plantlets were observed at 6 weeks of inoculation (Dasgupta et al., 2008).

### 2.4 Determination of growth parameters

The growth parameters, such as shoot length (SL), number of shoots (NOS), number of nodes (NON), number of leaves (NOL), leaf area (LA), shoot fresh weight (SFW), shoot dry weight (SDW), number of roots (NOR), root length (RL), root fresh weight (RFW), and root dry weight (RDW), were determined under control (0 mM), 50 mM NaCl-, and 100 mM NaCl-mediated salinity stress conditions at 6 weeks after inoculation.

### 2.5 Antioxidative enzyme assay

For antioxidative enzyme assays [SOD, CAT, GPX, ascorbate peroxidase (APX), monodehydroascorbate reductase (MDAR), dehydroascorbate reductase (DHAR), glutathione reductase (GR), and polyphenol oxidase (PPO)], the frozen leaf sample of 6-week-old *in vitro* cultures (0.25 g) was ground in a pre-chilled mortar and pestle with liquid nitrogen and homogenized with 2.5 mL of extraction buffer containing 50 mM sodium phosphate buffer (NaH_2_PO_4_, pH 7.8), 1 mM EDTA, 0.1% Triton X-100, 1 mM ascorbate, and 10% sorbitol. The homogenates were centrifuged at 15,000 rpm at 4°C for 20 min. The supernatant was used for all the enzyme assays (Dasgupta et al., 2008).

### 2.6 SOD and CAT activities

The ability of the nitroblue tetrazolium chloride (NBT) reactions to be inhibited was used to estimate SOD activities (EC 1.15.1.1). The reaction mixture (1.5 mL) contained 50 mM sodium phosphate buffer (NaH_2_PO_4_, pH 7.8), 1 mM Ethylenediaminetetraacetic acid (EDTA), 13 mM methionine, 75 µM NBT, and 50 μL enzyme extract. Riboflavin (2 µM) was added to the reaction mixture and illuminated with 20-W fluorescence tubes for 15 min. Non-illuminated tubes without an enzyme extract served as the control. Absorbance was recorded at 560 nm using a UV–visible spectrophotometer (Thermo Fisher Scientific, Waltham, MA, USA), and the unit of SOD enzyme that inhibits 50% NBT was expressed as U g^−1^ FW (Giannopolitis and Ries, 1977).

Similarly, the rate at which H_2_O_2_ is scavenged, as indicated by a decrease in absorbance at 240 nm, was used to calculate CAT activity (EC 1.11.1.6). The reaction mixture (1.5 mL) contained 100 mM sodium phosphate buffer (NaH_2_PO_4_, pH 7.0), 60 mM H_2_O_2_, and 50 μL enzyme extract. The decreased H_2_O_2_ was monitored at 240 nm for 1 min, and the molar extinction coefficient (40 mM^−1^ cm^−1^) of CAT was used to quantify its activity (µM min^−1^ g^−1^ FW) using the Aebi (1983) method.

### 2.7 GPX and APX activities

The production of tetraguaiacol was observed to estimate GPX activity (EC 1.11.1.7) using the extinction coefficient (26.6 mM^−1^ cm^−1^) (Urbanek et al., 1996). The reaction mixture (2.0 mL) contained 100 mM sodium phosphate buffer (NaH_2_PO_4_, pH 7.0), 0.1 mM EDTA, 5.0 mM guaiacol, 15 mM H_2_O_2_, and 50 μL enzyme extract. The increase in absorbance was recorded at 470 nm for 1 min. The enzyme activity was quantified by the amount of tetraguaiacol formed using its molar extinction coefficient (26.6 mM mM^−1^ cm^−1^). The results were expressed as µmol guaiacol min^−1^ g^−1^ FW, taking into consideration that 4 mol of H_2_O_2_ was reduced to produce 1 mol of tetraguaiacol.

Similarly, the molar extinction coefficient (2.8 mM^−1^ cm^−1^) was used to quantify APX activity (EC 1.11.1.1), which was measured by monitoring the decrease in absorbance at 290 nm brought on by ascorbate oxidation and expressed as µmol ascorbate oxidized min^−1^ g^−1^ FW (Nakano and Asada, 1981).

### 2.8 MDAR, DHAR, and GR activities

MDAR (EC 1.6.5.4) was calculated using Hossain and Asada’s (1984) method. The reaction mixture (3 mL) contained 50 mM sodium phosphate buffer (NaH_2_PO_4_, pH 7.0), ascorbate (0.5 mM), H_2_O_2_ (0.1 mM), NADPH (0.1 mM), and enzyme extract (0.2 mL). The addition of NADPH started the reaction, and the consumption of NADPH was monitored by a 1-min decrease in absorbance at 340 nm using an extinction coefficient of 6.22 mM^−1^ cm^−1^ (Krivosheeva et al., 1996). MDAR was expressed as µM NADPH min^−1^ (1 unit) g^−1^ FW.

Oxidized ascorbate was catalyzed to ascorbate by DHAR (EC 1.8.5.1). The DHAR activity was assessed using the extinction coefficient (2.8 mM^−1^ cm^−1^) (Nakano and Asada, 1981). For the assay of this enzyme, the reaction mixture (1 mL) contained 0.7 mL of phosphate buffer (50 mM, pH 7.0) with EDTA (0.1 mM), 0.1 mL of reduced glutathione (2.5 mM) in phosphate buffer, 0.1 mL of dehydroascorbate (DHA; 2 mM), and 0.1 mL of enzyme extract. To prevent fast oxidation at room temperature, DHA was freshly prepared and kept on ice until it was added to the reaction mixture in the cuvette. The increase in absorbance at 290 nm was recorded after the addition of DHA, which indicated the formation of ascorbate. The enzyme activity was expressed as µM min^−1^ (1 unit) g^−1^ FW of leaf tissue, taking 2.8 mM^−1^ cm^−1^ as the absorbance coefficient of ascorbate (Krivosheeva et al., 1996).

The GR (EC 1.6.4.2) activity was assayed in the reaction mixture (1 mL) containing 0.86 mL of oxidized glutathione (1 mM), 0.1 mL of NADPH (2 mM), and 0.04 mL of enzyme extract. GR activity was estimated using the extinction coefficient (6.22 mM^−1^ cm^−1^) after the rate of NADPH oxidation decreased in absorbance at 340 nm for 2 min and expressed as µM min^−1^ (1 unit) g^−1^ FW (Cakmak et al., 1993).

### 2.9 PPO activities

The PPO activity of the healthy leaves was determined following the methodology of Mukherjee and Ghosh (1975). The reaction mixture was composed of 2.5 mL of sodium phosphate buffer (0.1 M, pH 7.0) and 0.3 mL of 0.01 M catechol (by dissolving in 0.1 M phosphate buffer, pH 7.0). The reaction was started by the addition of 0.2 mL of enzyme extract, and the increase in absorbance between 0 and 60 s of incubation was recorded at 420 nm. PPO activity was expressed as increments in absorbance at 420 nm per minute and milligrams of protein during the first 1 min, the period in which the enzymatic activity is linear, and expressed as U min^−1^ g^−1^ FW (Belles et al., 2006).

### 2.10 Statistical analyses

The experiment was set up in a two-way factorial completely randomized design (fCRD) replicated thrice with duplicate determinations. The experiment was repeated twice, and the pooled data were presented. Growth parameters and antioxidant activities were recorded under control (0 mM) and NaCl-mediated salinity stress conditions (50 and 100 mM) at 6 weeks of inoculation. The stress index (SI) was estimated following the formula [SI = (Treatment − Control)/Control × 100]. Statistical analyses were performed using the analysis of variance (ANOVA), with square root transformation applied where necessary (Gomez and Gomez, 1984). Pearson’s correlation coefficient, principal component analysis (PCA), and genotype-by-trait hierarchical clustering were performed using Python 3.13.2, an open-source software tool.

## 3 Results

### 3.1 *In vitro* growth performances of sweet potato nodal cultures under NaCl-mediated salinity stress conditions

**Figure 1:**
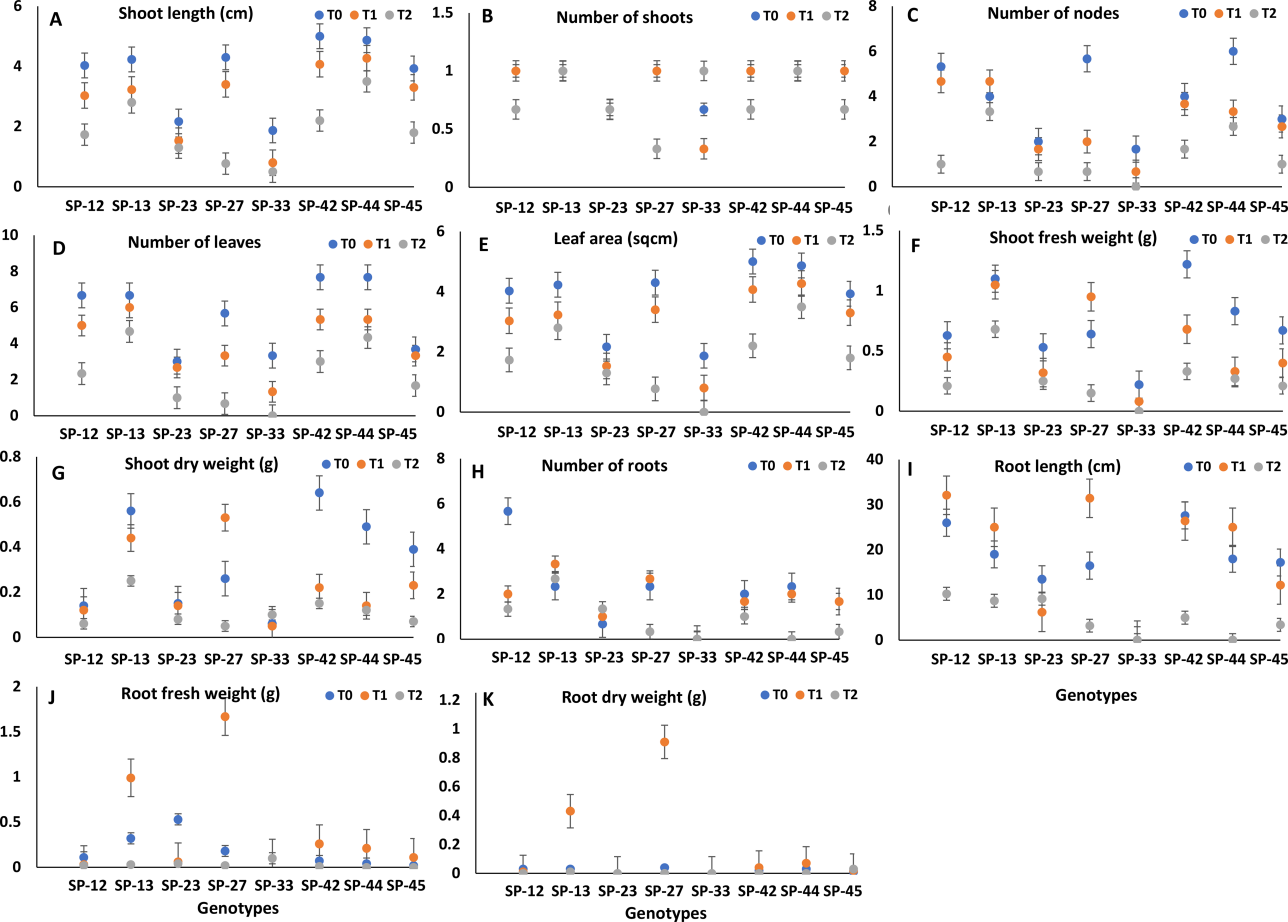
**(A–K)** Effect of NaCl-mediated salinity stress conditions (T_0_, 0 mM; T_1_, 50 mM; T_2_, 100 mM) on growth performances of eight sweet potato genotypes *in vitro*. Values are the pool of two experiments with three replicates and two determinations, and bars represent the standard error of means. **(A)** Shoot length (SL), **(B)** number of shoots (NOS), **(C)** number of nodes (NON), **(D)** number of leaves (NOL), **(E)** leaf area (LA), **(F)** shoot fresh weight (SFW), **(G)** shoot dry weight (SDW), **(H)** number of roots (NOR), **(I)** root length (RL), **(J)** root fresh weight (RFW), and **(K)** root dry weight (RDW).

**Table 1** depicts the level of significance among the growth parameters, such as SL, NOS, NON, NOL, LA, SFW, SDW, NOR, RL, RFW, and RDW, from the nodal cultures at different levels of salinity *in vitro*. The growth parameters significantly declined with increased levels of salinity (50 and 100 mM NaCl) compared to their control (**Figures 1A–K**).

**Table 1:** ANOVA (mean sum of squares) for different growth parameters of sweet potato genotypes under NaCl-mediated salinity stress conditions *in vitro*.

Source	df	SL	NOS	NON	NOL	LA	SFW	SDW	NOR	RL	RFW	RDW
Genotypes (G)	7	9.89**	0.15^NS^	12.81**	25.04**	14.04**	0.57^NS^	0.14^NS^	8.31**	458.35**	0.46^NS^	0.11^NS^
Salinity (S)	2	24.91**	0.18^NS^	40.54**	66.88**	32.29**	1.32^NS^	0.34^NS^	10.05**	1,510.91**	1.05^NS^	0.25^NS^
G × S	14	0.77*	0.13^NS^	2.62**	1.39^NS^	4.54**	0.08^NS^	0.04^NS^	2.78**	121.34**	0.32^NS^	0.10^NS^
Error	48	1.05	0.12	1.20	2.83	1.02	0.04	0.01	0.75	30.59	0.02	0.002

G, Genotype; S, salinity; *df*, degrees of freedom; SL, shoot length; NOS, number of shoots; NOL, number of leaves; LA, leaf area; SFW, shoot fresh weight; SDW, shoot dry weight; NOR, number of roots; RL, root length; RFW, root fresh weight; RDW, root dry weight.

^*^Significance at p ≤ 0.05.

^**^Significance at p ≤ 0.01.

^NS^Non-significant.

#### 3.1.1 *In vitro* organogenesis

Shoot length ranged from 1.87 cm (SP-33) to 5.00 cm (SP-42) under control conditions (T_0_), 0.8 cm (SP-33) to 4.27 cm (SP-44) at 50 mM NaCl, and 0.77 cm (SP-27) to 3.5 cm (SP-44) at 100 mM NaCl (**Figure 1A**). SP-33 could not survive at 100 mM NaCl; however, SP-44 maintained shoot length throughout the treatments. All the genotypes maintained one shoot per plantlet throughout the treatments, hence showing statistical non-significance (**Figure 1B**). However, SP-13 and SP-44 maintained significantly good shoot health and leaf retention in terms of NON, NOL, LA, SFW, and SDW at control, 50 mM, and 100 mM NaCl treatments (**Figures 1C–G**). The highest shoot fresh weight was observed in SP-42 (1.22 g) under control conditions, which decreased to 0.33 g at 100 mM NaCl. Similarly, shoot dry weight was the highest in SP-42 (0.64 g) under control conditions, but reduced to 0.15 g at 100 mM NaCl. Genotypes SP-12, SP-44, and SP-45 also retained higher shoot fresh and dry weights under stress.

#### 3.1.2 Rooting responses

The root attributes significantly decreased under NaCl-induced salinity when compared with the control. SP-12 exhibited the highest number of roots (5.67) and root length (32.1 cm) under control conditions, but both parameters were reduced under stress, with only 1.33 roots and 10.26-cm root length at 100 mM NaCl. The root fresh weight declined substantially under salinity stress compared to the control in all the tested genotypes. SP-13 exhibited higher root fresh weight (0.03 g) at 100 mM NaCl, whereas SP-33 and SP-45 showed the lowest values (0.01 and 0.02 g, respectively), indicating poor performance under salinity conditions (**Figures 1H–K**).

SP-12, SP-13, and SP-23 possessed higher NOR, RL, RFW, and RDF at higher doses (100 mM NaCl) (**Figures 1H–K**). Although SP-44 exhibited better shoot growth at a higher level of NaCl (100 mM), the rooting response was poor in this genotype. SP-13 and SP-23 exhibited higher shoot and root growth at 100 mM salinity stress.

### 3.2 Enzymatic antioxidant activities of sweet potato nodal cultures under NaCl-mediated salinity stress conditions *in vitro*

**Figure 2:**
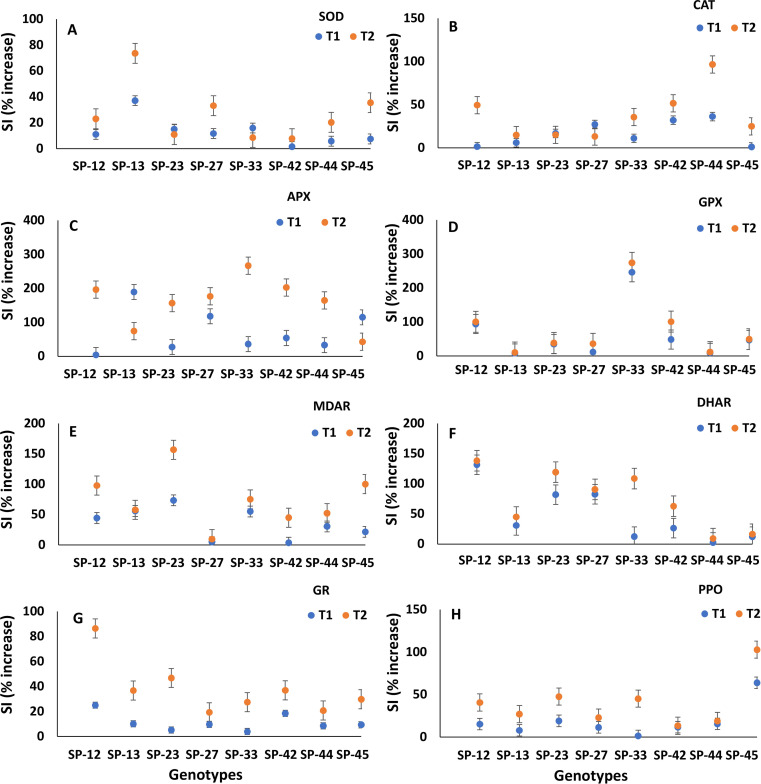
**(A–H)** Stress index (SI; % increase) for the antioxidative activities. **(A)** Superoxide dismutase (SOD), **(B)** catalase (CAT), **(C)** ascorbate peroxidase (APX), **(D)** guaiacol peroxidase (GPX), **(E)** monodehydroascorbate reductase (MDAR), **(F)** dehydroascorbate reductase (DHAR), **(G)** glutathione reductase (GR), and **(H)** polyphenol oxidase (PPO) of eight sweet potato genotypes under *in vitro* NaCl-mediated salinity stress conditions (T_1_, 50 mM; T_2_, 100 mM) compared to control (T_0_, 0 mM). Values are the pool of two experiments with three replicates and two determinations, and bars represent the standard error of means.

**Table 2** indicates the level of significance among the antioxidative enzyme properties, such as SOD, CAT, APX, GPX, MDAR, DHAR, GR, and PPO, in the leaf tissues from the nodal cultures at different levels of salinity *in vitro*. The induction of antioxidative enzymes significantly increased among the genotypes with increased levels of salinity (50 and 100 mM NaCl) compared to their control (**Figures 2A–H**).

**Table 2:** ANOVA (mean sum of squares) for antioxidative enzymes in leaf tissues of sweet potato genotypes under NaCl-mediated salinity stress conditions *in vitro*.

Source	df	SOD	CAT	APX	GPX	MDAR	DHAR	GR	PPO
Genotypes (G)	7	12,931,993.0^**^	5,956.3^**^	898,596.9^NS^	22,550,886.9^**^	87,704.2^**^	302,414.9^NS^	78,191.3^**^	0.202^*^
Salinity (S)	2	68,521,294.8^**^	1,824.4^**^	4,408,287.3^*^	19,459,770.5^**^	85,006.2^**^	3,152,228.6^**^	159,615.9^**^	0.813^**^
G × S	14	4,569,412.3^**^	358.1^NS^	466,373.6^NS^	2,214,770.0^**^	6,351.4^NS^	185,007.2^NS^	4,309.1^NS^	0.035^NS^
Error	48	1,121,778.1	167.8	866,478.9	176,186.3	9,083.4	145,567.6	14,740.5	0.079

G, genotype; S, salinity; *df*, degrees of freedom; SOD, superoxide dismutase; CAT, catalase; APX, ascorbate peroxidase; GPX, guaiacol peroxidase; MDAR, monodehydroascorbate reductase; DHAR, dehydroascorbate reductase; GR, glutathione reductase; PPO, polyphenol oxidase.

^*^Significance at p ≤ 0.05.

^**^Significance at p ≤ 0.01.

^NS^Non-significant.

#### 3.2.1 SOD and CAT activities

SOD activity in stress-free control plantlets was in the range of 9,856.2 (SP-13) to 15,725.4 U g^−1^ FW (SP-33). The activity under NaCl-mediated salinity conditions was increased (over control) to 13,509.8 in SP-13 to 18,234.8 in SP-33 at 50 mM NaCl, and 15,055.3 in SP-23 to 19,435.4 in SP-27 at 100 mM NaCl. SOD increased by 1.6%–37.1% in 50 mM NaCl and 7.8%–73.6% in 100 mM NaCl compared to the stress-free control (**Figure 2A**). SP-13, SP-45, and SP-27 exhibited a higher increase in SOD activities at 100 mM NaCl.

The hydrogen peroxide-scavenging CAT activity was enhanced in all studied genotypes; however, the magnitude of the increment varied among the genotypes. CAT activity (µM min^−1^ g^−1^ FW) in leaves was in the range of 20.3 (SP-23) to 69.4 (SP-44) in stress-free control conditions, 23.7 (SP-23) to 94.4 (SP-44) at 50 mM NaCl, and 23.4 (SP-23) to 136.4 (SP-44) at 100 mM NaCl. Under higher NaCl stress conditions (100 mM NaCl), SP-44, SP-42, SP-12, and SP-33 were found to have higher CAT activities (**Figure 2B**).

#### 3.2.2 APX and GPX activities

APX was enhanced in *in vitro* NaCl-stressed sweet potato leaves compared to the control. APX (µM ascorbate oxidized min^−1^ g^−1^ FW) under control conditions was in the range of 321.4 (SP-12) to 833.3 (SP-44), while it was 631.0 (SP-33) to 2,273.8 (SP-13) at T_1_ and 952.4 (SP-12) to 2,202.4 (SP-44) at T_2_. The increment rate in APX activity was the highest at 2.6-fold in SP-33, followed by 2.03-fold in SP-42 and 1.96-fold in SP-12 (**Figure 2C**).

Under NaCl stress conditions, GPX increased in all eight sweet potato genotypes compared to the control. GPX under control conditions was in the range of 1,097.7 to 4,497.9 µmol guaiacol min^−1^ g^−1^ FW, while it was 2,647.5 to 8,718.5 under 50 mM NaCl and 2,686.7 to 9,018.4 µmol guaiacol min^−1^ mg^−1^ protein under 100 mM NaCl-mediated salinity treatment *in vitro*. GPX increased 2.46-fold and 2.4-fold in SP-33 under 50 mM and 100 mM NaCl-mediated salinity conditions *in vitro* (**Figure 2D**).

#### 3.2.3 MDAR and DHAR activities

A significant increase in MDAR activity (µM min^−1^ g^−1^ FW) was observed among the studied genotypes. MDAR activity varied between 75.3 (SP-45) and 279.6 μM NADPH min^−1^ g^−1^ FW (SP-13) under control conditions. Under 100 mM NaCl treatment, MDAR was observed in the range of 91.4 (SP-45) to 435.5 (SP-13). The sweet potato genotype SP-23 highly induced MDAR at 0.73-fold at T_1_ and 1.57-fold at T_2_ (**Figure 2E**).

The pre-breeding sweet potato genotypes exhibited a significant increase in DHAR activity (µM min^−1^ g^−1^ FW) under NaCl-mediated stress conditions over the control. However, the genotypic variations within the treatment were observed as non-significant. DHAR was in the range of 714.3–1,428.6, 1,083.3–1,797.6, and 1,261.9–2,190.5 µM min^−1^ g^−1^ FW at T_0_, T_1_, and T_2_, respectively. The rate of increase in DHAR at 50 and 100 mM NaCl compared to the control was higher in SP-12 (1.32-fold and 1.38-fold, respectively) (**Figure 2F**).

#### 3.2.4 GR activities

GR activity (mM min^−1^ g^−1^ FW) varied significantly across the genotypes and treatments. GR activity was in the range of 295.7 to 551.1, 369.6 to 598.1, and 436.8 to 759.4 in the investigated genotypes at 0, 100, and 100 mM NaCl-mediated stress conditions, respectively. The magnitude of GR was registered as higher in SP-12, followed by SP-13 and SP-23, at both T_1_ and T_2_, compared to other genotypes (**Figure 2G**).

#### 3.2.5 PPO activities

The result revealed significant differences in PPO among the eight pre-breeding sweet potato genotypes under stress-free control and NaCl-mediated stress conditions. PPO activity in sweet potato leaves was 0.60–1.22 U min^−1^ g^−1^ FW under control conditions, 0.98–1.30 U min^−1^ g^−1^ FW in T_1_, and 1.15–1.77 U min^−1^ g^−1^ FW in T_2_. The increase in PPO was higher in SP-45 at T_1_ and T_2_ compared to the control (0.64-fold and 1.03-fold, respectively) (**Figure 2H**).

### 3.3 Correlation among the variables under NaCl-mediated salinity stress conditions *in vitro*

**Figure 3:**
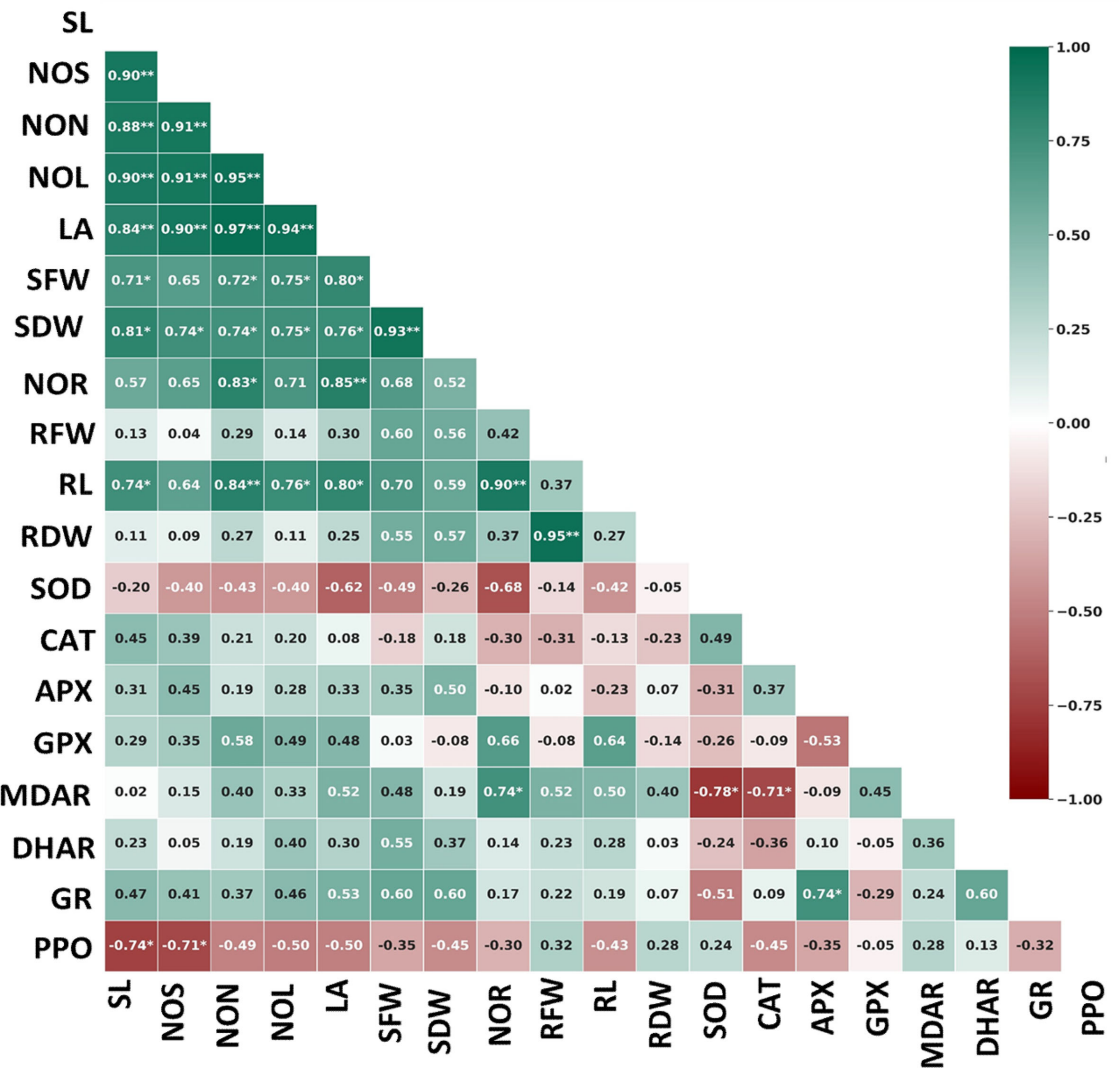
Pearson’s correlation coefficient (r value) for growth parameters and antioxidative enzymes in sweet potato genotypes under *in vitro* NaCl-mediated salinity stress conditions. The colour scheme from green (1.0) to red (−1.0) indicates positive and negative correlation. The threshold values (r) at p ≤ 0.05 and p ≤ 0.01 are 0.707 and 0.834 (*n* = 24), respectively. SL, shoot length; NOS, number of shoots; NON, number of nodes; NOL, number of leaves; LA, leaf area; SFW, shoot fresh weight; SDW, shoot dry weight; NOR, number of roots; RL, root length; RFW, root fresh weight; RDW, root dry weight; SOD, superoxide dismutase; CAT, catalase; APX, ascorbate peroxidase; GPX, guaiacol peroxidase; MDAR, monodehydroascorbate reductase; DHAR, dehydroascorbate reductase; GR, glutathione reductase; PPO, polyphenol oxidase.

**Figure 3** indicates significant relationships among various morpho-physiological properties and enzymatic antioxidant activities in eight pre-breeding sweet potato lines. Pearson’s correlation coefficients (r) matrix revealed a strong and positive integration among the growth parameters except in RFW and RDW, which were moderately correlated with SFW, SDW, and NOR. Among the antioxidative enzymes, APX (r = 0.74) and DHAR (r = 0.60) showed strong positive correlations with GR. MDAR was significantly and negatively correlated with SOD (r = −0.78) and CAT (r = −0.71). GPX strongly and positively correlated with NOR (r = 0.66) and RL (r = 0.64), whereas MDAR strongly correlated with NOR (r = 0.74). GR correlated positively with SFW (r = 0.60) and SDW (r = 0.60). SOD and PPO negatively correlated with all the growth parameters. PPO possessed a strong negative correlation with shoot parameters, SL (r = −0.74) and NOS (r = −0.71), in particular. However, SOD was negatively associated with the root induction, particularly with NOR (r = −0.68) (**Figure 3**). The correlation study indicated a positive influence of GR and GPX on shoot induction and GPX and MDAR on root induction. SOD and PPO negatively correlated with shoot and root organogenesis under NaCl-mediated salinity stress conditions.

### 3.4 Principal component analysis among the sweet potato genotypes and variables under NaCl-mediated salinity stress conditions *in vitro*

**Figure 4:**
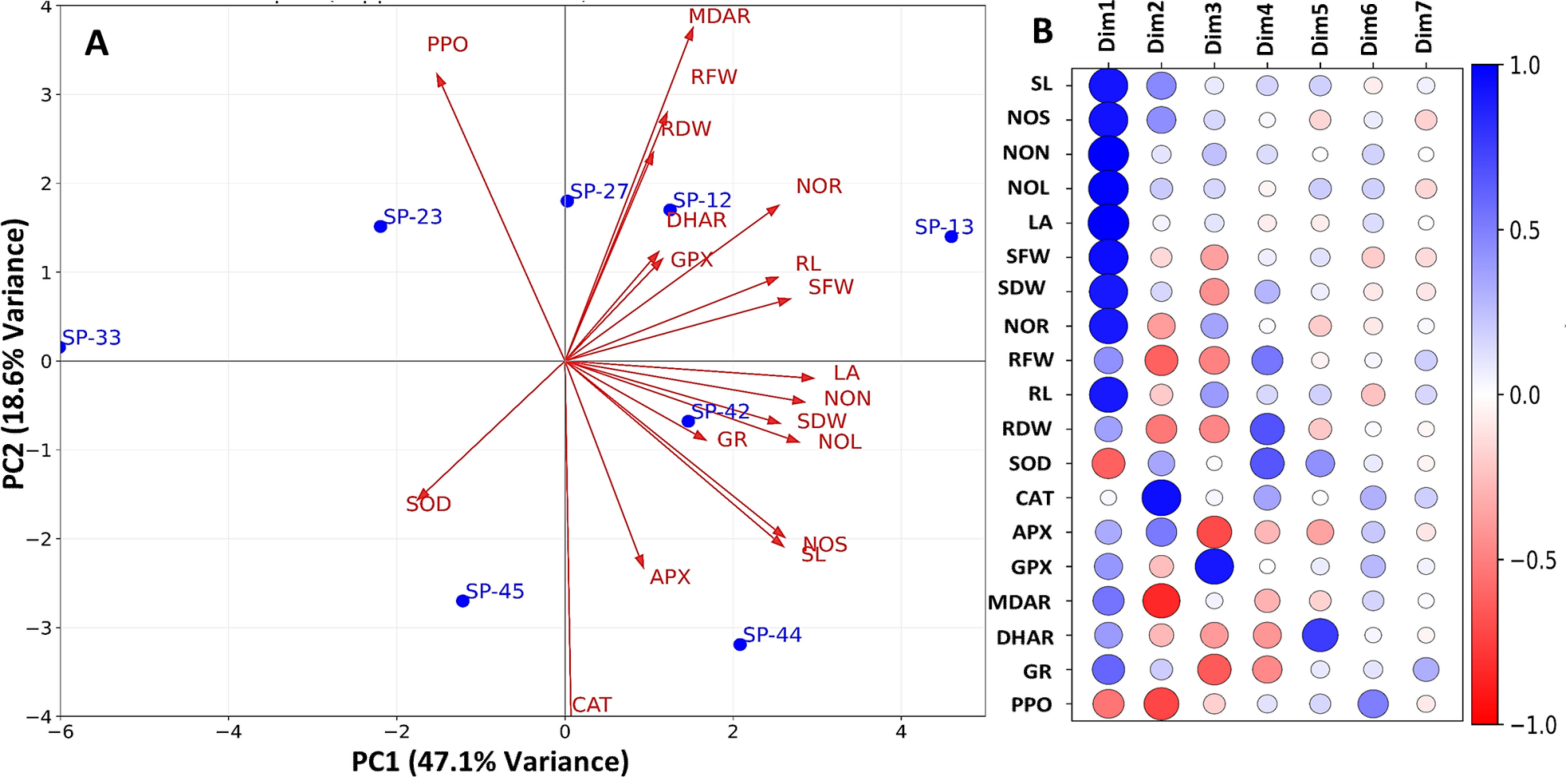
**(A)** Principal component analysis (PCA) for growth parameters and antioxidative enzymes in sweet potato genotypes under *in vitro* NaCl-mediated salinity stress conditions. **(B)** Correlation plot of the variables versus the principal components. SL, shoot length; NOS, number of shoots; NON, number of nodes; NOL, number of leaves; LA, leaf area; SFW, shoot fresh weight; SDW, shoot dry weight; NOR, number of roots; RL, root length; RFW, root fresh weight; RDW, root dry weight; SOD, superoxide dismutase; CAT, catalase; APX, ascorbate peroxidase; GPX, guaiacol peroxidase; MDAR, monodehydroascorbate reductase; DHAR, dehydroascorbate reductase; GR, glutathione reductase; PPO, polyphenol oxidase.

PCA visualized the relationships among the eight sweet potato pre-breeding lines, growth parameters, and antioxidative enzyme properties under *in vitro* NaCl-mediated salinity conditions (**Figures 4A, B**). Among the seven principal components, the first two PCs explain 65.7% of the total variance in the data. PC1 accounts for 47.1% of the total variance, whereas PC2 contributes 18.6% of the variance (**Figure 4A**). The visualization depicts how different sweet potato genotypes relate to each other and their significance towards NaCl-mediated salinity stress tolerance concerning their growth performances and antioxidative enzyme properties. The PCA implies that genotype SP-13, followed by genotypes SP-12 and SP-42, is closely related to higher growth performances and antioxidative enzymes (MDAR, DHAR, GR, and GPX). Sweet potato genotypes SP-33, SP-23, and SP-45 were negatively related to the growth performances attributed to SOD, CAT, and PPO. **Figure 4B** indicates the principal component-wise (Dim1–Dim7) relationship among different variables in sweet potato nodal cultures under *in vitro* stress conditions. In PC1 (Dim1), all the variables, including antioxidative enzymes (AOEs), showed a positive association with the growth performances of sweet potato *in vitro*, except SOD and PPO, which were negatively associated with plantlet growth. CAT showed a neutral response in Dim1, which was strongly and negatively correlated in Dim2. Similarly, GPX was highly and negatively related to the *in vitro* plantlet growth. Overall, SP-13 excelled in terms of growth performances and MDAR, DHAR, GR, and GPX under *in vitro* NaCl-mediated salinity stress conditions.

### 3.5 Hierarchical clustering among the sweet potato genotypes and variables under NaCl-mediated salinity stress conditions *in vitro*

**Figure 5:**
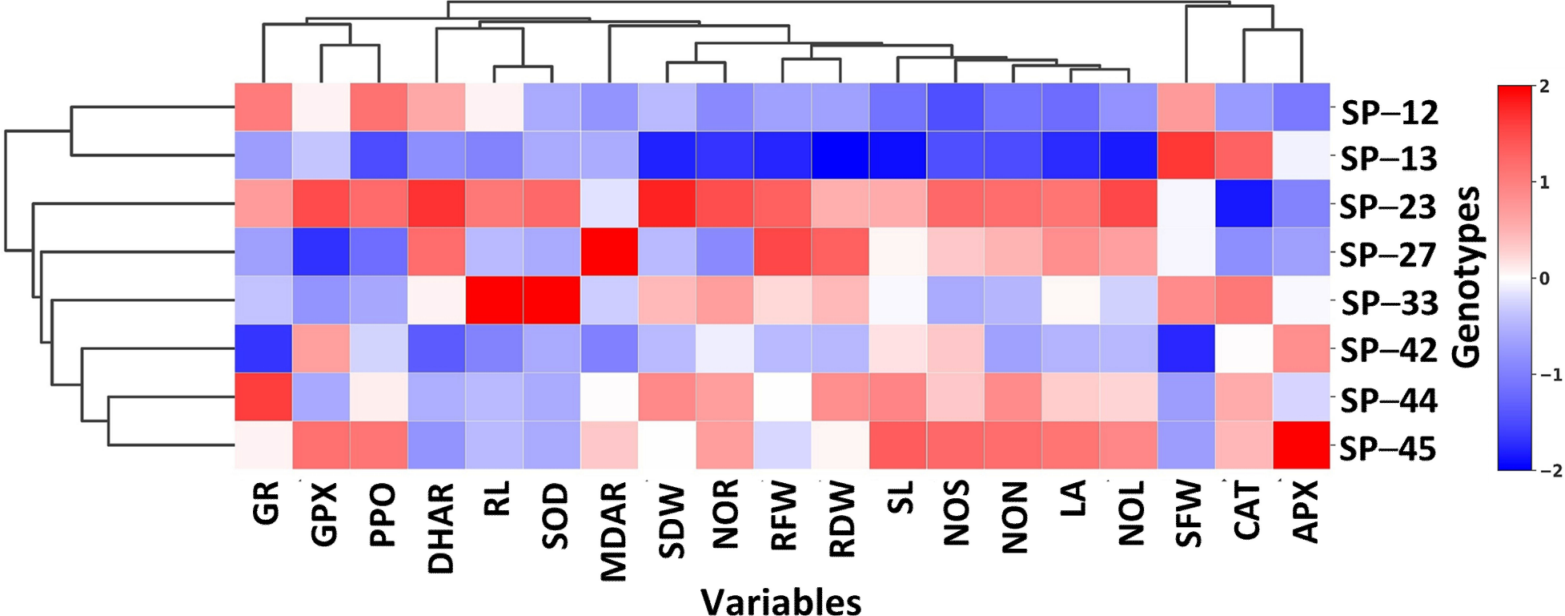
Hierarchical clustering of the sweet potato genotypes, growth parameters, and antioxidative enzymes under *in vitro* NaCl-mediated salinity stress conditions. SL, shoot length; NOS, number of shoots; NON, number of nodes; NOL, number of leaves; LA, leaf area; SFW, shoot fresh weight; SDW, shoot dry weight; NOR, number of roots; RL, root length; RFW, root fresh weight; RDW, root dry weight; SOD, superoxide dismutase; CAT, catalase; APX, ascorbate peroxidase; GPX, guaiacol peroxidase; MDAR, monodehydroascorbate reductase; DHAR, dehydroascorbate reductase; GR, glutathione reductase; PPO, polyphenol oxidase.

Genotypes-by-trait hierarchical clustering (**Figure 5**) distinctly grouped the genotypes into four major clusters. The blue color indicates a stress tolerance index, whereas the red color indicates susceptibility. Cluster I, including SP-12 and SP-13, showed tolerance to salinity stress with respect to all the variables except for SOD and PPO. SP-23 in cluster II exhibited high susceptibility to salinity. Cluster III, with two subgroups, included five moderately tolerant genotypes. Similarly, the variables were grouped into four major clusters. Clusters I and II exhibited the shoot growth attributed to AOEs APX, GR, and DHAR. However, clusters III and IV included root attributes complemented by AOEs GPX, MDAR, CAT, SOD, and PPO. The hierarchical clustering revealed that the genetic pattern of the genotypes varied significantly with respect to the growth attributes and AOEs to withstand salinity stress conditions. SP-13 and SP-12 exhibited tolerance, whereas SP-33, SP-23, and SP-45 showed susceptibility to NaCl-mediated salinity stress.

## 4 Discussion

A reliable screening technique is the primary concern when selecting breeding populations for salinity stress tolerance (Mondal et al., 2022). Field screening of closely related pre-breeding populations often confuses breeders due to inconsistent edaphic situations, adverse biotic factors, and environmental influences (Tao et al., 2021). A rapid *in vitro* screening and selection procedure is the most convenient tool (Raoufi et al., 2021) to authenticate the field evaluation results of the pre-breeding populations. In the present study, we assessed eight pre-breeding sweet potato genotypes for *in vitro* NaCl-mediated salinity stress conditions to authenticate our field evaluation results and further forward the lines for augmenting future breeding strategies for salinity tolerance.

Selection breeding approaches are easier, more reliable, and more efficient in selecting and developing plants for salinity stress (Dasgupta et al., 2007). An *in vitro* selection process using nodal segment, shoot apex culture, or callus culture provides a meaningful tool for stringent screening, selection, and development of stress-tolerant plants. The nodal segment culture is often used for *in vitro* salinity tolerance studies due to lower possibilities of somaclonal variations (Mukherjee, 2001). Dasgupta et al. (2008) and Paulino and Mari (2016) conducted *in vitro* salinity tolerance studies using nodal cultures with NaCl (0.25%–1.5%) and concluded that the NaCl dose beyond 0.5% (100 mM) is detrimental for shoot and root organogenesis. Hence, we considered 0.25% (50 mM) and 0.5% (100 mM) for our *in vitro* screening study to rapidly screen the pre-breeding sweet potato lines for salinity stress tolerance.

NaCl-mediated salinity hinders plant growth and development due to Na^+^ ion imbalance, leading to oxidative damage (Hasanuzzaman et al., 2021). High salt concentration inhibits shoot and root organogenesis, thereby decreasing the fresh and dry biomass. Shoot and root induction are highly affected due to Na^+^ ion disruption, cellular toxicity, and ROS overproduction in the nodal cultures. The plant adapts to salinity through avoidance mechanisms such as maintaining growth and development (Tao et al., 2021). Similarly, tolerance mechanisms encompass biochemical and molecular events, as well as antioxidative enzyme activities (Dasgupta et al., 2007, 2008; Saleem et al., 2024). High salt concentration has a detrimental effect on shoot and root induction, resulting in decreased shoot and root biomass. Reduction in shoot growth exhibited as a rapid response to increased osmotic pressure and a slower response due to the accumulation of Na^+^ in leaf tissues (Munns and Tester, 2008). Plant adaptation to salinity involves osmotic tolerance, Na^+^ exclusion, and tissue tolerance, as evidenced by better organogenesis in the tolerant genotypes. Previous reports have suggested a significant decrease in shoot number as the salinity level rose, which may have been caused by salt’s detrimental effects on bud formation and differentiation (Roussos et al., 2006). In our study, SL, NON, NOL, LA, NOR, and RL significantly decreased among the sweet potato genotypes with increased NaCl concentrations. Under stress-free MS medium, shoot and root organogenesis were achieved in all the genotypes except delayed rooting in SP-33. Shoot growth falls significantly when NaCl is increased to a threshold level of 40 mM, which creates an osmotic pressure of 0.2 MPa (Munns and Tester, 2008). Khenifi et al. (2011) found that the supply of NaCl adversely affects the shoot length of potatoes and that the concentration and exposure duration of salt directly correlate with the negative impact on plantlet growth.

According to earlier findings from the shoot apex growth test, rooting was one of the factors most impacted by salt in tomato cultivars (Mercado et al., 2000), potatoes (Khenifi et al., 2011), and mulberries (Vijayan et al., 2003). In the present study, *in vitro* shoot and root growth were significantly affected at 50 and 100 mM NaCl concentrations. SP-13 and SP-12 consistently maintained shoot and root growth *in vitro*. Although shoot organogenesis was better in SP-44 at higher NaCl concentrations (100 mM), rooting responses were very poor. In sweet potato, nodal cuttings are used for plant propagation. Thus, salinity tolerance studies in nodal explants and their organogenesis are essentially important to derive salt-tolerant plants.

The shoot and root inhibition were due to Na^+^ and Cl^−^ ion toxicity. Na^+^ evidenced more toxicity than Cl^−^ during the immediate effect of salt stress (Munns and Tester, 2008). Ion toxicity results in osmotic stress leading to ROS overproduction, which restricts the nutrient availability and mobility in the plant cell essential for organogenesis and causes poor shoot and root growth (Mondal et al., 2022). Moreover, osmotic stress has an immediate effect on organogenesis over the ionic stress caused by salinity (Munns and Tester, 2008). Rapid cell proliferation impaired osmotic stress, cellular ion toxicity, and ROS accumulation by inducing AOE mechanisms. The genotypes that induced shoot organogenesis under adverse NaCl stress conditions hold the genetic potential to overcome ion toxicity-led ROS overproduction by deploying a strong AOE network and are considered to have salinity tolerance (Mondal et al., 2023). AOEs participated in ROS-scavenging actions in early and late events and were termed the first line of AOEs and secondary or subsequent lines of AOEs, respectively (Wahengbam et al., 2023; Iqbal et al., 2024).

Salt stress manifests oxidative stress mediated by ROS, and plant adaptive mechanisms strategically equilibrate the negative consequences of ion toxicity-led ROS overproduction by deploying AOE machinery (Acosta–Motos et al., 2017). ROS removal by an array of cellular antioxidative enzymes mechanistically prevents cellular toxicity, DNA, RNA, protein, and membrane oxidation, collectively called oxidative stress (Mittler, 2017). SOD, CAT, and GPX are the primary defense enzymes induced in the plant cell under stress conditions that scavenge ROS (Mondal et al., 2023). SOD catalyzes the dismutation of superoxide ion radical (O_2_^•−^) into H_2_O_2_ and molecular O_2_. CAT and GPX scavenge the extricated H_2_O_2_ into H_2_O and molecular oxygen (O_2_) (Ighodaro and Akinloye, 2018). In the subsequent events, APX acts as an electron donor in the free-radical detoxification process to compensate for ROS-impaired cellular damage. MDAR and DHAR are crucial AOEs that recycle ascorbic acid by accelerating the ascorbate–glutathione (AsA–GSH) cycle (Foyer and Noctor, 2011). Critically, MDAR reduces intermediate monodehydroascorbate, and DHAR eliminates the fully oxidized form of dehydroascorbate. GR recycles oxidized glutathione back to its reduced form (Kumara et al., 2024). Polyphenols are a group of metabolites reported to have a stringent role in *in vitro* growth and development, which are strategically being scavenged by PPO (Sahoo et al., 2009). In redox biology, rapid oxidation and reduction of Cys residues are eventually linked with the rapid accumulation of ROS scavengers (Mittler, 2017). ROS detoxification mechanisms influence the chloroplast regulatory regimes to protect the photosystem (Deinlein et al., 2014). Better detoxification and antioxidative enzymes are a genotypic response, which are more prominent in tolerant genotypes than in susceptible ones (Munns and Tester, 2008).

In the present study, NaCl (50 and 100 mM) significantly induced the AOEs (0.77–2.6-fold) in the *in vitro* nodal cultures of the eight sweet potato pre-breeding lines compared to the stress-free control. The rate of AOE accumulation was higher in the tolerant genotypes than in the susceptible ones. Genotypes SP-13, SP-12, and SP-42 exhibited a higher stress tolerance index (STI) for enzymatic antioxidative mechanisms than the susceptible ones to cope with the salinity stress. Shoot and root induction are also strongly correlated with the AOE accumulation as early and late events. Devi et al. (2020) and Sahoo et al. (2018) established a strong and positive correlation of AOEs with biotic and abiotic stress tolerance, as demonstrated by PCA and genotype-by-trait hierarchical clustering. From the present study, it is evident that the SOD and CAT participated in ROS scavenging at the early stage, thus negatively correlated with the growth parameters. However, the accumulation of late events in GR and APX was attributed to shoot organogenesis, which signifies eliminating the negative consequences of ion toxicity and ROS molecules (Sahoo et al., 2020). GPX, MDAR, and DHAR exhibited root organogenesis, indicating reduced ascorbate’s influence on the rooting. The principal component analysis indicated the association of APX and GR with SL, NOS, NON, NOL, LA, SFW, and SDW. Higher growth rate and dry matter production are attributed to salt stress tolerance in halophyte plants (Flowers and Colmer, 2008). Genotypes SP-13 and SP-42 showed prominent shoot organogenesis under *in vitro* NaCl-mediated salinity stress conditions. However, SP-13 and SP-12 possessed commendable root organogenesis with higher MDAR, DHAR, and GPX correlated with NOR, RL, RFW, and RDW.

Pearson’s correlation coefficients (r) matrix revealed that APX (r = 0.74) and DHAR (r = 0.60) showed strong positive correlations with GR. PPO possessed a strong negative correlation with shoot parameters SL and NOS, indicating its association with shoot retardation. Interestingly, the hierarchical clustering distinctly grouped the tolerant and susceptible genotypes into three clusters. However, the growth parameters and AOEs were grouped into four major clusters based on their contribution to *in vitro* organogenesis. The heat map and hierarchical clustering are meaningful tools for understanding the relationships among and with the genotypes and variables. Sweet potato genotypes SP-13, SP-12, and SP-42 demonstrated better shoot and root organogenesis under salinity stress. We correlated the *in vitro* organogenesis with the AOE accumulations from the nodal cultures. The early defense enzymes, such as SOD and CAT, participated in impairing the negative consequences of the NaCl in the growth medium at the early stage and thus could not correlate positively with the growth variables. However, MDAR, DHAR, GPX, and GR strongly and positively correlated with all the growth parameters.

Plants under salt stress have higher levels of these antioxidative enzymes, and there is a relationship between salt tolerance and these enzyme levels (Mittova et al., 2003; Saleem et al., 2024). According to Dasgupta et al. (2008), salt-stressed plants’ leaves exhibited higher levels of enzymes CAT, GPX, and SOD than control plants. The tolerant genotype displayed a more pronounced increase than the susceptible genotype. Thamodharan and Arumugam Pillai (2014) reported the role of antioxidative enzyme activity in salt stress and salinity screening in rice through callus culture. Accordingly, the findings imply that CAT and GPX activities, in conjunction with SOD activity, are crucial for protecting against O_2_^•−^ and H_2_O_2_ scavenging (Mittova et al., 2003) and that the active participation of these enzymes is connected, at least in part, to sweet potato plants’ ability to withstand oxidative stress caused by salt. The findings here are consistent with reports that salt stress increases the activity of antioxidative enzymes. This lends credence to the theory that the scavenging of ROS provides a mechanism of tolerance to the transient salt stress through increased activity of AOEs in leaves of the sweet potato plantlets. The potential role of AOEs in sweet potato to salt stress would shed light on the molecular processes underlying salt-induced oxidative stress in plants. Furthermore, a detailed salt stress signal transduction study would lay out a better understanding of the signaling pathways in sweet potato against salinity stress (Zhu, 2002). Apparent Na^+^ uptake needs further investigation to understand enigmatic ion transport effects in salt-tolerant sweet potato. Considering better growth performances and antioxidative mechanisms, genotypes SP-13, SP-12, and SP-42 may be forwarded for further evaluation under natural salinity regimes under field conditions for yield and yield-attributing characteristic assessment.

## 5 Conclusion

NaCl-mediated salinity stress had a detrimental effect on *in vitro* nodal cultures and their antioxidant properties in sweet potato. We have demonstrated the efficient induction of AOEs in impairing the growth and development of nodal cultures in eight contrasting sweet potato pre-breeding lines. The genotypes displayed varied growth responses from the nodal cultures under NaCl-mediated salinity stress conditions, which determine their genetic potential to tolerate the salinity threshold. A strong correlation was observed among the growth parameters of nodal cultures with AOE induction, which was validated via PCA and hierarchical clustering. The second or subsequent lines of defense enzymes, APX and GR, scavenge the stress consequences effectively in the tolerant lines, which enabled better shoot organogenesis and proliferation under stress. Similarly, GPX, MDAR, and DHAR correlated with better root induction under salinity. SP-13 outstandingly responded to the *in vitro* NaCl stress, maintaining growth performances and AOE properties. Our results clarified the confounding factors among the pre-breeding sweet potato lines orchestrated with AOEs, leading to NaCl-mediated salinity stress tolerance. The study manifested a quick, efficient, and reliable screening tool to assess genetic resources to augment salinity stress tolerance selection breeding strategies. The tool can also be used to quickly assess and select the best pre-breeding lines prior to release, following the variety release procedures. In future lines of study, a detailed *in vivo* evaluation of tolerant genotypes SP-13, SP-12, and SP-42 under salinity conditions would provide a concise understanding of Na^+^/Cl^−^/K^+^ markers on genotype × environment interactions leading to ROS–AOE cross-talk to derive stress-tolerant plants.

## Data Availability

The original contributions presented in the study are included in the article/supplementary material. Further inquiries can be directed to the corresponding authors.
